# Elucidation of the synergistic action of *Mentha Piperita* essential oil with common antimicrobials

**DOI:** 10.1371/journal.pone.0200902

**Published:** 2018-08-01

**Authors:** Antonio Rosato, Alessia Carocci, Alessia Catalano, Maria Lisa Clodoveo, Carlo Franchini, Filomena Corbo, Giuseppe Gerardo Carbonara, Antonio Carrieri, Giuseppe Fracchiolla

**Affiliations:** 1 Department of Pharmacy–Drug Sciences, University of Bari “A. Moro”, Bari, Italy; 2 Department of Agro-Environmental and Territorial Sciences, University of Bari “A. Moro”, Bari, Italy; Jamia Millia Islamia, INDIA

## Abstract

*Mentha piperita* L. essential oil (EO) is employed for external use as antipruritic, astringent, rubefacient and antiseptic. Several studies demonstrated its significant antiviral, antifungal and antibacterial properties. The aim of this work is the study of the synergistic effects of *M*. *piperita* EO with antibacterials and antifungals that are widely available and currently prescribed in therapies against infections. The observed strong synergy may constitute a potential new approach to counter the increasing phenomenon of multidrug resistant bacteria and fungi. *In vitro* efficacy of the association *M*. *piperita* EO/drugs was evaluated against a large panel of Gram-positive and Gram-negative bacteria and yeast strains. The antimicrobial effects were studied by checkerboard microdilution method. The synergistic effect of *M*. *piperita* EO with gentamicin resulted in a strong growth inhibition for all the bacterial species under study. The synergistic effect observed for *M*. *piperita* EO and antifungals was less pronounced.

## Introduction

Bacterial resistance to antibiotic therapy is a growing emergency [1]. In the last years, several research programs have focused on designing new compounds possessing potential antimicrobial activity in order to avoid this problem [2–6]. New sources, especially plant-derived antimicrobial compounds, have been extensively studied in recent years [7,8].

Plant essential oils (EOs) have been examined in detail for their pharmacological properties and may constitute a promising source for new natural drugs [9–14]. Currently, approximately 3000 EOs are known of which 300 are commercially available in the agronomic, alimentary, sanitary and pharmaceutical fields [15–19]. Plant-derived EOs are natural mixtures of a certain complexity. At times, they may contain more than 100 components at quite different concentrations. These components encompass two groups of distinct biosynthetic origin: the main group includes terpenes and terpenoids, while the other one includes aromatic and aliphatic constituents with low molecular weight [20]. Plausibly, the presence of all these compounds in the EOs explains the absence of microbial resistance or adaptation to their pharmacological properties. Thus, EOs constitute an effective alternative or complementary therapy to synthetic compounds, without manifesting the same side effects [21].

### Properties and uses of *M*. *piperita L*.

*M*. *piperita* L. (Lamiaceae) is used as raw material in several different applications in foods and cosmetics; leaves and flowers are used for medicinal preparations [22].

*M*. *piperita* EO is used in perfume industry, cosmetics, aromatherapy, spices, nutrition, etc. Several studies have shown the antiviral, antibacterial, antifungal properties and antioxidant activities of EOs and of the extract of the herbal parts of *M*. *piperita* obtained through different preparative procedures [23]. As the chemical profile of *M*. *piperita* EO depends on both the method used for the extraction and the amount of molecules extracted, it is very difficult to establish which specific biological target is responsible for the action. The biological profile of the EO may be the result of a synergistic action of all the molecules contained in the EO or it may reflect only the activity of the main molecules.

### Chemical composition and potential therapeutic applications of *M*. *piperita L*. EO

The content of EOs can be detected by hyphenated gas chromatography with mass spectrometry (GC/MS) technique [24,25]. *M*. *piperita* EO is composed by monoterpenes, menthone, menthol and their derivatives. Several authors have underlined the role of EOs in the management of several therapeutic conditions, as inflammation of the oral mucosa, irritable colon, spastic discomfort of the upper gastrointestinal tract and bile ducts, catarrhs of the respiratory tract. A therapeutic approach based on a combination of drugs could contribute to overcoming antibiotic resistance [26–28].

Recently, we assessed and reported the positive synergism against *Candida* spp. of the echinocandin anidulafungin combined with aspirin or with other NSAIDs [29,30]. All these observations prompted us to investigate the combination of commercially available *M*. *piperita* EO with well-known synthetic antimicrobials, with the aim of providing a greater effectiveness to combat infections and overcome the phenomenon of drug resistance [31–35].

### Aims of research

Gentamicin and ampicillin were chosen as antibacterial agents, while amphotericin B was chosen as antifungal. Gentamicin is an aminoglycoside antibiotic largely used for the treatment and prevention of severe Gram-negative bacterial infections. However, its severe side effects (oto- and nephrotoxicity) limit its use. In addition, psychiatric symptoms related to gentamicin (confusion, anorexia, depression, disorientation and visual hallucinations) may occur.

Ampicillin is a *β*-lactam antibiotic that showed several side effects. Recently, an increased resistance to this antibiotic has been reported [36].

Amphotericin B is a polyene antifungal drug which is considered the drug of choice for the treatment of mycosis; it is often combined with azoles. However, several authors have observed that in the last decades *Candida* species have become resistant to treatment to azoles alone and to azoles in association with amphotericin B [37].

In the class of azoles, fluconazole and miconazole were chosen for the association with *M*. *piperita* EO. Regarding all this, the aim of the present study was to examine the possible synergistic effect of *M*. *piperita* EO with common antimicrobials.

We recently reported the *in vitro* synergistic activity of certain combinations of essential oils with antimicrobials [38–40]. The antimicrobial activity of the *M*. *piperita* EO against different Gram-positive and Gram-negative bacteria and fungi, along with its synergistic effects when combined with antimicrobial drugs (gentamicin, ampicillin, amphotericin B, miconazole and fluconazole), has been studied by following the microdilution checkerboard method. The composition of commercially available *M*. *piperita* EO used in our experiments has been confirmed by GC/MS analyses [41–45].

## Materials and methods

### Microorganisms

Ten bacterial strains from American Type Culture Collection (ATCC, Rockville, MD, USA) were used for tests: *Bacillus cereus ATCC 10876*, *Bacillus subtilis ATCC 6633*, *Staphylococcus aureus ATCC 6538p*, *Staphylococcus aureus ATCC 29213*, *Staphylococcus aureus ATCC 43300 (MRSA)*, *Enterococcus faecalis ATCC 29212*, *Escherichia coli ATCC 25922*, *Klebsiella pneumoniae ATCC 19883*, *Acinetobacter baumanni ATCC 19606*, *Pseudomonas aeruginosa ATCC 27853*. Seven Candida species from ATCC were used for antifungal tests: *Candida albicans ATCC 10231*, *Candida albicans ATCC 24433*, *Candida albicans ATCC 90028*, *Candida guilliermondii ATCC 6260*, *Candida glabrata ATCC 15126*, *Candida krusei ATCC 14243*, *Candida kefyr ATCC 204093*.

### Microbial and yeast cultures

The bacterial species were cultured on Mueller Hinton agar (MHA, Oxoid) and each bacterial suspension was composed of 2–3 colonies for each strain taken from an MHA plate and dissolved in 2 mL of MHB (Mueller Hinton Broth). The resulting suspensions were diluted with 0.85% NaCl solution and then adjusted to 1x10^8^ CFU/mL (0.5 McFarland).

The fungal strains were subcultured twice on Sabouraud dextrose agar before being tested. Yeast cells were washed four times in sterile saline. Each fungal suspension was taken from its frozen stock at –70°C. The strains were inoculated in 5 mL of Sabouraud dextrose broth, and then incubated under stirring at 35°C for 48 h.

### MIC evaluation protocols

MIC values were determined by broth microdilution method, in accordance with CLSI (Clinical and Laboratory Standards Institute) Protocol M07-A9 guidelines for bacteria and Protocol M27-A3 guidelines for yeasts [46,47].

### Antimicrobial activity

For antibacterial tests, a stock solution (EO/Ethanol 1:2.5, 40% v/v with Tween 80, 0.1%) was diluted 1:20 in MHB to obtain a 2% (v/v) final solution. Doubling dilutions of the EO from 2% to 0.015% for EO were prepared directly in 96 well microtiter trays in MHB. After the addition of 0.02 mL of the inoculum, the microtiter trays were incubated at 36°C for 24 h. The final concentration of ethanol was 1.5% (v/v). The MHB medium 0.1% (v/v) Tween 80 and ethanol 1.5%, (without EO) was used as a positive growth control.

Preparation of the EO for the antifungal tests followed the same procedure as the one for the antibacterial tests. A small quantity of inoculum was dissolved in RPMI 2% glucose and then spectrophotometrically adjusted to 0.5 x 10^3^ to 2.5 x 10^3^ CFU/mL (McFarland, turbidity standard). The initial inocula were confirmed by plating serial dilutions and determining the colony counts. A total of 0.1 mL of each yeast suspension was dispensed into serially diluted wells containing the drugs or the EO, achieving final drug concentration. After the addition of 0.1 mL of inoculum, the plates were incubated at 36°C for 48 h. MIC was defined as the lowest concentration of the mixtures at which no visible growth of the fungal strains could be detected compared to their growth in the negative control well. MIC values are given in mg/mL and μg/mL for *M*. *piperita* EO and antimicrobial drugs, respectively.

MIC determinations were realized in triplicate in three independent assays.

### FICI determination

MIC data of the antimicrobial compounds and *M*. *piperita* EO were converted into Fractional Inhibitory Concentration (FIC), determined using the formula FIC = (MIC_A_^combination^/MIC_A_^alone^). MIC values for the EO-drugs associations were defined as the lowest concentration at which no visible growth of the microbial strains could be detected compared to their growth in the control well, as described in Eucast document [48].

### Microdilution checkerboard method

In the combination assays, the checkerboard procedure described by White *et al*. [49] was followed to evaluate the synergistic action of the EO with selected drugs. Twelve double serial dilutions of the EO were prepared following the same method used to evaluate the MIC. Dilutions of the EO were prepared together with a series of double dilutions of the antimicrobial drugs: for antifungal drugs in the range of 32.0–0.66 μg/mL, for antibacterial in the range of 64.0–0.125 μg/mL and for *M*. *piperita* EO in the range of 18.2–0.09 mg/mL.

This method was used to mix each antimicrobial compounds dilution with the appropriate concentrations of EO in order to obtain a series of concentration combinations of the EO with each particular drug. In our experimental protocol, the substance combinations were analysed by calculating the FIC index (FICI) as follows: FIC of the EO plus FIC of the drug. Generally, the FICI value was interpreted as: i) a synergistic effect when ≤ 0.5; ii) an additive or indifferent effect when > 0.5 and <1; iii) an antagonistic effect when > 1 [49]. The concentrations prepared accounted for 40%, 20%, 10%, and 5% of the MIC value for the EO, and 25%, 12.5%, 6.25%, 3.12% of the MIC value for the antibiotic. Also, the combination of two components is shown graphically in a Cartesian diagram by applying the isobole method. The non-interaction of the two components results in a straight line, whereas the occurrence of an interaction is shown by a concave isobole [50–53].

### Chemicals and materials

Antifungal and antibacterial agents Gentamicin, Ampicillin, Amphotericin B, Miconazole and Fluconazole were purchased from Sigma S.r.l. (Milan, Italy). Commercially available pure *M*. *piperita* EO (Lot 140/0000324, 10.2018, 10 mL) was provided by Erbenobili S.r.l. (Corato, Bari,—Italy). C7-C30 alkanes mixture and solvents, all of analytical grade, were purchased from Sigma Aldrich S.r.l. (Milan, Italy), filters were supplied by Agilent Technologies Italia S.p.a. (Milan, Italy).

### Gas chromatography–mass spectrometry equipment

The gas chromatographic analyses have been performed on HP GC/MS 6890N-5973N MSD HP ChemStation equipped with autosampler and HP-5MS column (crosslinked 5% PH ME siloxane) 30 m x 0.25 mm x 0.25 μm Film Thickness. The following temperature program was applied: 40°C (4 min), 4°C per minute heating up to 280°C (30 min). The mass spectrometer was operated in EI mode at 70 eV; the ion source temperature was 220°C. The mass spectra were measured in the range of 35–360 amu.

### Compound identification

For chemical characterization, a standard solution of 100 μL of the pure EO in 1 mL of ethyl acetate was prepared. The solution was filtered and 1 μL was analyzed by GC/MS. The sample was analyzed in triplicate. Qualitative analysis was executed comparing the calculated Linear Retention Indices (LRI) and Similarity Index Mass Spectra (SI/MS) for the obtained peaks with the analogous data from NIST2011 and Adams 4th ed. (2007) databases. LRI of each compound was obtained by temperature programming analysis and was determined in relation to an homologous series of *n*-alkanes (C7–C30) under the same operating conditions. LRI was calculated following the Van den Dool and Kratz equation [24,25,45,54] and compared with the Arithmetic Index (AI) from NIST2011 database and Adams, 4th ed. (Adams 2007). SI/MS were determined as reported by Koo *et al*. [55]. Component relative percentages were calculated on the basis of GC peak areas without using correction factors.

## Results and discussion

The present research has tested *M*. *piperita* EO in association with several antifungal and antibacterial drugs. The effects have been evaluated on ten strains of Gram-positive and Gram-negative bacteria and seven strains of *Candida* spp. FICI values for *M*. *piperita* EO in combination with antibacterial agents as gentamicin and ampicillin are reported in **Table 1**.

**Table 1:** *Mentha piperita* EO and antibacterial drugs–fractional inhibitory concentration (FIC) and FIC indices (FICI).

	Gentamicin				Ampicillin			
	MICo	MICc	FIC	FICI	MICo	MICc	FIC	FICI
*Bacillus cereus ATCC* 10876								
Drug (μg/ml)	2.00±0.58	0.06±0.02	0.03	0.08	1.00±0.48	0.06±0.02	0.06	0.46
EO (mg/ml)	4.55±1.32	0.23±0.13	0.05		4.55±1.32	1.82±0.79	0.40	
*Bacillus subtilis ATCC* 6633								
Drug (μg/ml)	0.50±0.48	0.01±0.02	0.02	0.07	0.12±0.08	0.01±0.02	0.08	0.13
EO (mg/ml)	4.55±1.32	0.23±0.13	0.05		4.55±1.32	0.23±0.13	0.05	
*Staphylococcus aureus ATCC* 6538p								
Drug (μg/ml)	2.0±0.58	0.06±0.02	0.03	0.103	0.12±0.08	0.03±0.02	0.24	0.44
EO (mg/ml)	9.10±2.63	0.91±0.39	0.10		9.10±2.63	1.82±0.79	0.20	
*Staphylococcus aureus ATCC* 29213								
Drug (μg/ml)	0.5±0.58	0.06±0.02	0.12	0.22	1.0±0.29	0.06±0.02	0.06	0.46
EO (mg/ml)	4.55±2.63	0.46±0.12	0.10		4.55±2.63	1.82±0.79	0.4	
*Staphylococcus aureus ATCC* 43300								
Drug (μg/ml)	8.0±2.31	2.0±0.58	0.23	0.30	8.0±2.31	2.0±0.58	0.25	0.65
EO (mg/ml)	9.10±2.63	0.46±0.12	0.05		9.10±2.63	3.64±1.05	0.40	
*Enterococcus faecalis ATCC* 29212								
Drug (μg/ml)	8.0±2.31	1.0±0.29	0.12	0.32	1.0±0.48	0.06±0.02	0.06	0.46
EO (mg/ml)	9.10±2.63	1.82±0.79	0.20		9.10±2.63	3.64±0.66	0.40	
*Escherichia coli ATCC* 25922								
Drug (μg/ml)	1.0±0.48	0.03±0.02	0.03	0.43	16.0±4.62	4.0±2.31	0.50	0.55
EO (mg/ml)	9.10±2.63	3.64±1.05	0.40		9.10±2.63	2.27±0.66	0.05	
*Klebsiella pneumoniae ATCC* 19833								
Drug (μg/ml)	32.0±9.24	1.0±0.29	0.03	0.43	16.0±4.62	4.0±2.31	0.25	0.50
EO (mg/ml)	9.10±2.63	3.64±1.05	0.40		9.10±2.63	2.27±0.66	0.25	
*Acinetobacter baumannii ATCC* 19606								
Drug (μg/ml)	8.00±2,31	0.5±0.14	0.06	0.46	16.0±4.62	4.0±2.31	0,25	0,50
EO (mg/ml)	9.10±2.63	3.64±1.05	0.40		9.10±2.63	2.27±0.66	0.25	
*Pseudomonas aeruginosa ATCC* 27853								
Drug (μg/ml)	2.00±0.58	0.06±0.01	0.03	0.08	16.0±4.62	4.0±2.31	0.23	0.50
EO (mg/ml)	9.10±2.63	0.46±1.05	0.05		9.10±2.63	2.27±0.98	0.23	

MICo ± S.E.M. = MIC of an individual sample; MICc ± S.E.M. = MIC of an individual sample at the most effective combination; FIC = fractional inhibitory concentration; FICI = FIC of antibiotic + FIC of *M*. *piperita* EO.

FICI values for the association with gentamicin and ampicillin were, respectively, in the range of 0.07–0.46 and 0.13–0.65 against all tested bacterial strains. It is also interesting to underline that the MIC value for gentamicin is markedly reduced when combined with *M*. *piperita* EO, as the MIC value for this particular association was found to be more than 30-fold lower against six out of the ten bacteria strains considered. The most interesting result was obtained for *Bacillus subtilis* ATCC 6633 for which the MIC value of gentamicin was found to have decreased from 0.5 to 0.01 μg/mL (FICI = 0.07). Moreover, a promising result was obtained also against *Acinetobacter baumannii* ATCC 19606, a Gram-negative bacillus resistant to treatment with gentamicin alone. In this case, a 16-fold reduction of gentamicin MIC (FICI = 0.46) was observed when used with *M*. *piperita* EO. The strong synergy observed between gentamicin and the EO against the Gram-negative *Pseudomonas aeruginosa* ATCC 27853 and *Klebsiella pneumoniae ATCC* 19833 is worthy of note. In particular, the MICc value for gentamicin is much lower than that normally required to achieve the direct inhibition of bacterial growth (MICc: 0.06 μg/mL *vs* MICo: 2 μg/mL, MICc: 1 μg/mL *vs* MICo: 32 μg/mL). Associations of ampicillin with *M*. *piperita* EO showed a marked synergistic effect against *Escherichia coli ATCC* 25922 (FICI = 0.08) and *Bacillus subtili*s ATCC 6633 (FICI = 0.13). Results for the associations with antifungals are reported in **Table 2**.

**Table 2:** *Mentha piperita* EO and antifungal drugs–fractional inhibitory concentration (FIC) and FIC indices (FICI).

	Amphotericin B				Fluconazole				Miconazole			
Candida Strains (ATCC)	MICo	MICc	FIC	FICI	MICo	MICc	FIC	FICI	MICo	MICc	FIC	FICI
*Albicans ATCC 10231*												
Drug (μg/ml)	1.00±0.29	0.06±0.02	0.06	0.46	1.00±0.29	0.06±0.02	0.06	0.46	2.00±0.58	0.13±0.04	0.06	0.46
EO (mg/ml)	2.28±1.30	0.91±0.26	0.40		2.28±1.30	0.91±0.26	0.40		2.28±1.30	0.91±0.26	0.40	
*Albicans ATCC 24433*												
Drug (μg/ml)	1.00±0.58	0.06±0.02	0.06	0.46	1.00±0.29	0.06±0.02	0.06	0.46	2.00±0.58	0.12±0.03	0.06	0.16
EO (mg/ml)	2.28±1.30	0.91±0.26	0.40		2.28±1.30	0.91±0.26	0.40		2.28±1.30	0.23±0.07	0.10	
*Albicans ATCC 90028*												
Drug (μg/ml)	1.00±0.29	0.06±0.02	0.06	0.46	1.00±0.29	0.06±0.02	0.06	0.46	1.00±0.29	0.40±0.30	0.40	0.46
EO (mg/ml)	2.28±1.30	0.91±0.26	0.40		2.28±1.30	0.91±0.26	0.40		2.28±1.30	0.14±0.33	0.06	
*Guilliermondii ATCC 6260*												
Drug (μg/ml)	1.00±0.29	0.06±0.02	0.06	0.46	2.28±1.30	0.25	0.06	0.46	0.50±0.14	0.10±0.03	0.20	0.25
EO (mg/ml)	2.28±0.66	0.91±0.26	0.40		2.28±1.30	0.91±0.26	0.40		2.28±0.66	0.12±0.03	0.05	
*Glabrata ATCC 15126*												
Drug (μg/ml)	2.00±0.58	0.50±0.14	0.25	0.30	16±4.620	1.00±0.29	0.06	0.47	1.00±0.29	0.06±0.02	0.06	0.46
EO (mg/ml)	1.14±0.66	0.06±0.02	0.05		1.14±0.66	0.46±0.45	0.40		1.14±0.66	0.46±0.35	0.40	
*Krusei ATCC 6258*												
Drug (μg/ml)	1.00±0.29	0.06±0.02	0.06	0.46	16±4.620	1.00±0.29	0.06	0.46	1.00±0.29	0.06±0.02	0.06	0.46
EO (mg/ml)	4.54±1.30	1.82±1.05	0.40		4.54±1.30	1.82±1.05	0.40		4.54±1.30	1.82±1.05	0.40	
*Kefyr ATCC 204093*												
Drug (μg/ml)	2.00±0.5	0.12±0.03	0.06	0.46	2.28±1.30	0.25±0.08	0.06	0.46	0.50±0.14	0.03±0.05	0.06	0.46
EO (mg/ml)	4.54±1.30	1.82±1.05	0.40		4.54±1.30	1.82±1.05	0.40		4.54±1.30	1.82±1.05	0.40	
*Parapsilosis ATCC 22019*												
Drug (μg/ml)	1.00±0.58	0.03±0.05	0.06	0.46	1.00±0.29	0.06±0.02	0.06	0.46	2.00±0.58	0.13±0.04	0.06	0.46
EO (mg/ml)	2.28±1.30	0.12±0.03	0.40		2.28±1.30	0.91±0.26	0.40		2.28±1.30	0.91±0.26	0.40	
*Tropicalis ATCC 750*												
Drug (μg/ml)	2.00±0.50	0.06±0.02	0.06	0.46	1.00±0.29	0.06±0.02	0.06	0.46	2.00±0.58	0.12±0.03	0.06	0.16
EO (mg/ml)	4.54±1.30	0.91±0.26	0.40		2.28±1.30	0.91±0.26	0.40		2.28±1.30	0.23±0.07	0.10	

MICo ± S.E.M. = MIC of an individual sample; MICc ± S.E.M. = MIC of an individual sample at the most effective combination; FIC = fractional inhibitory concentration; FICI = FIC of antifungal + FIC of *M*. *piperita* EO.

**Fig 1:**
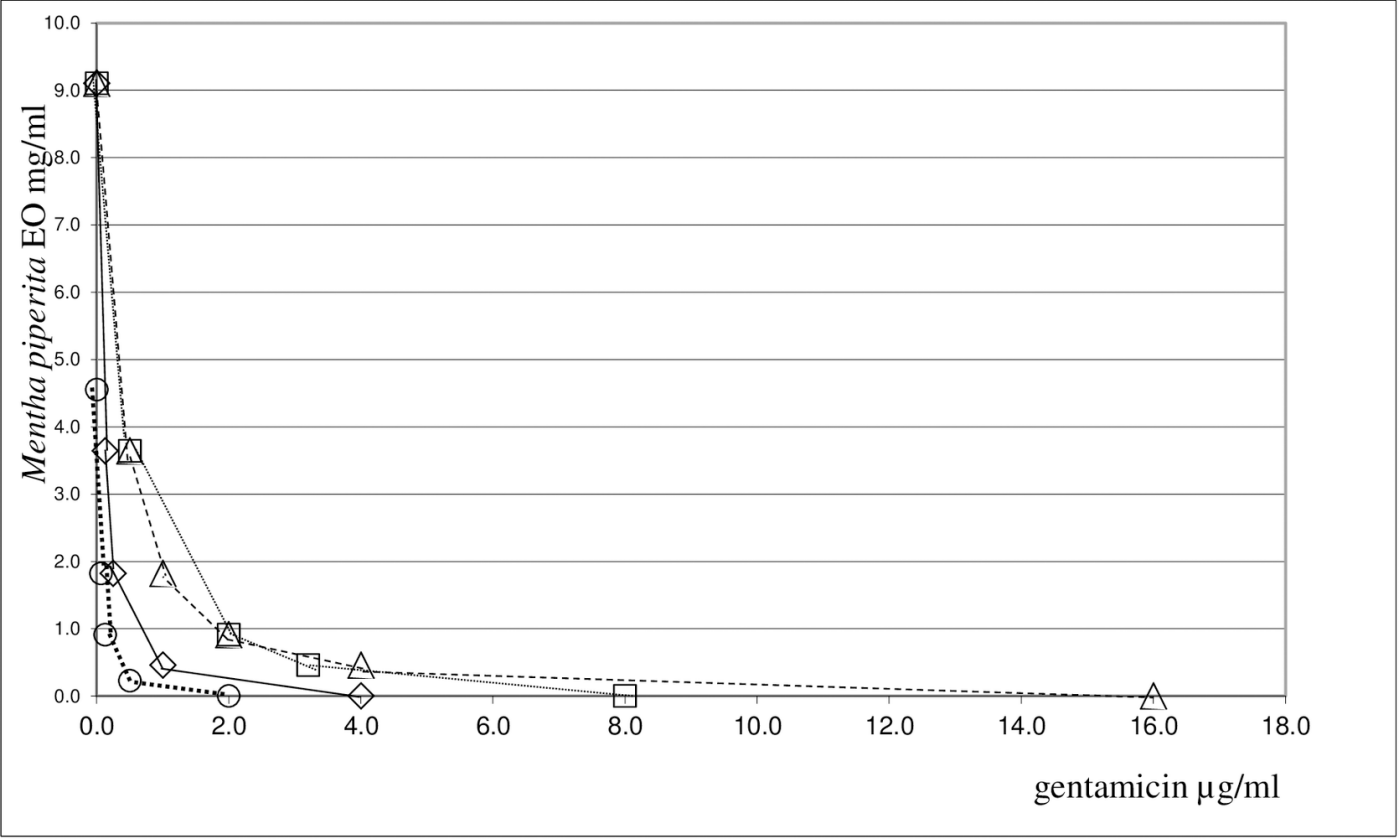
Isobole curves revealing the synergistic effect of *Mentha piperita* EO with gentamicin in inhibiting four bacterial strains. ◇ *P*. *aeruginosa ATCC 27853*, □ *E*. *faecalis ATCC 29212*, △ *K*.*pneuomoniae ATCC 13883*, ○ *B*.*cereus ATCC 10876*.

**Fig 2:**
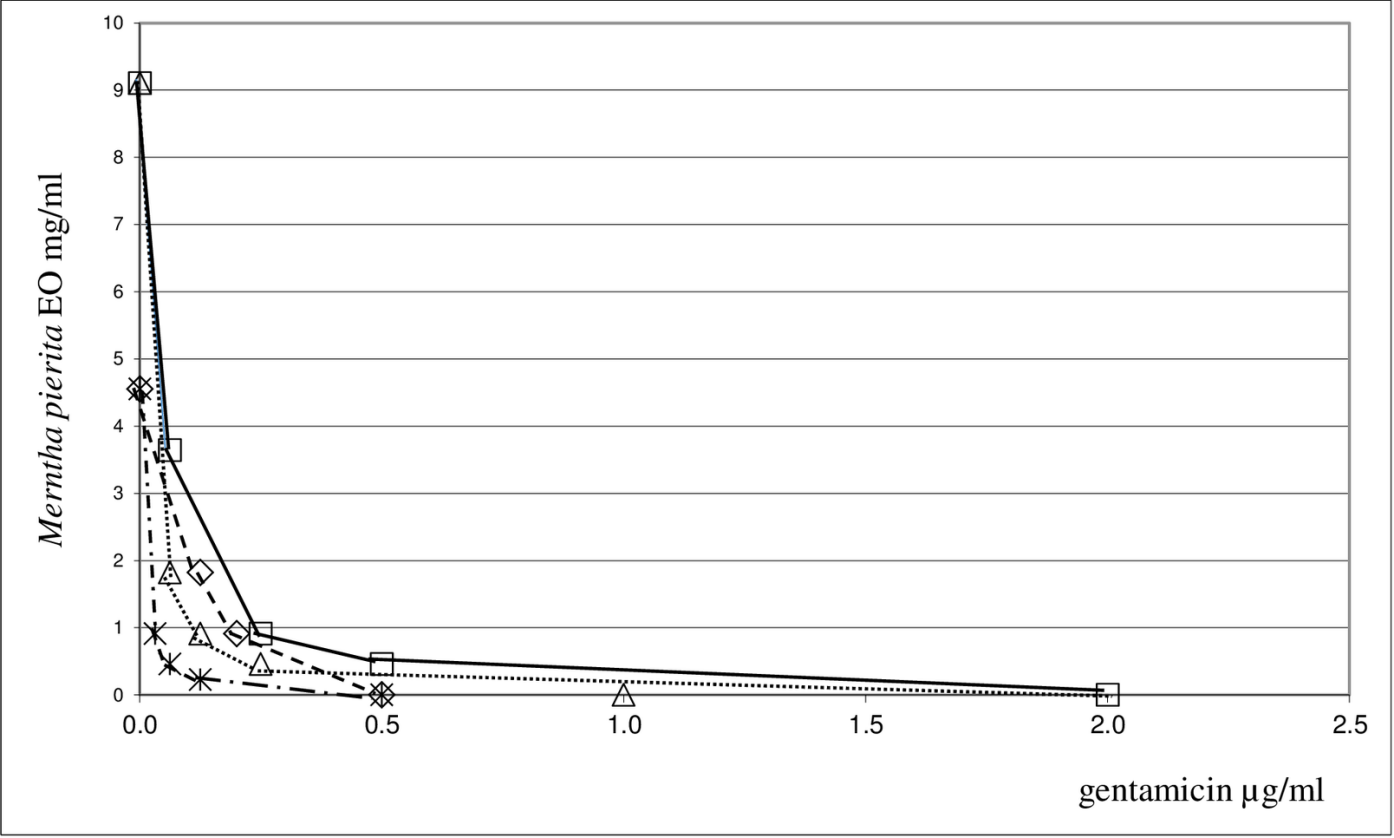
Isobole curves revealing the synergistic effect of *Mentha piperita* EO with gentamicin in inhibiting four bacterial strains. Δ *E*. *coli ATCC 25922*, ✳ *S*. *aureus ATCC 29213*, ◇ *B*. *subtilis ATCC 6633*, □ *S*. *aureus ATCC 6538P*.

**Fig 3:**
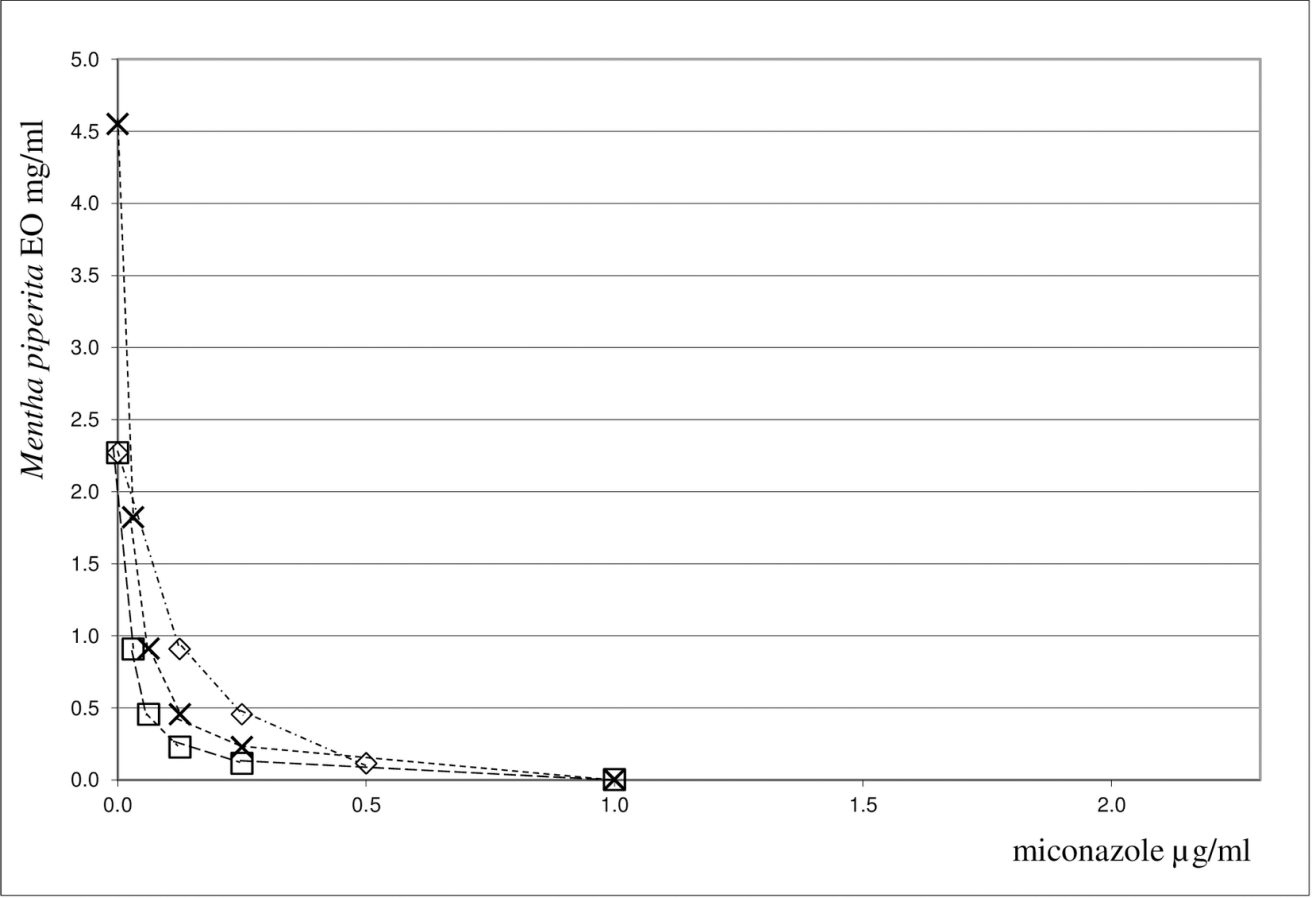
Isobole curves revealing the synergistic effect of *Mentha piperita* EO with miconazole in inhibiting three yeast strains. □ *Candida albicans ATCC 90028*, × *Candida krusei*, ◇ *Candida albicans ATCC 10231*.

A synergistic antifungal action was observed when *M*. *piperita* EO was combined with fluconazole, amphotericin B or miconazole against yeast strains under study. FICI values close to 0.4 were found for amphotericin B and fluconazole, whereas they ranged between 0.23 and 0.46 for miconazole. It is interesting to note that this association had a strong synergistic effect against the *C*. *albicans* and *C*. *guilliermondii* spp.. On the whole, the synergistic antimicrobial action demonstrated by combining *M*. *piperita* EO with antifungals was less pronounced against yeast strains than that of antibacterial agents combined with the same EO against bacteria strains. The synergistic interaction between *M*. *piperita* EO and the most promising antimicrobials, gentamicin and miconazole, is shown in **Figs 1–3**.

The combination of the two components is shown by an isobole method [50]. An isobole is an “iso-effect” curve, in which a combination of constituents is represented on a graph, whose axises represent the inhibitory doses of the individual agents. If the agents do not interact, the isobole (the line joining the points representing the combination to the points on the dose axises representing the individual doses with the same effect as the combination) will be a straight line. If the effect is additive, the curve of the isobole will be a “concave” line, thus indicating that the agents in the mixture are synergic. When the opposite occurs, a “convex” line will result, showing antagonism. In other words, the same biological effects of the isolated agents are obtained at lower (or higher) doses than those observed for the mixture. Our graphs indicate, indeed, a high synergism against all the bacteria and yeast strains examined. The synergistic or antagonistic relationship between antimicrobials may result from competition for a possible primary target [56]. Conversely, a synergistic multi-target effect may occur, involving enzymes, substrates, metabolites and proteins, receptors, ion channels, transport proteins, ribosomes, DNA/RNA and physicochemical mechanisms [40]. An alternative explanation may be that the interaction between different compounds may lead to changes in the structural conformation, and it may result in the reduction of the inhibitory activity. However, it is difficult to elucidate the exact mechanism of the synergistic effect without further investigation. In order to assess the impact of *M*. *piperita* EO in association with antimicrobials we evaluated the chemical composition of this EO by GC/MS analysis. For the chemical characterization of the commercially available EO used for the biological assay, GC/MS analysis were performed [42,43]. 27 components have been identified in the pure EO, 20 of which corresponded to 97.64% of the mixture. Menthol was found to be the major component, amounting to 68% of the mixture. The composition of the EO has been summarized in **Table 3**.

**Table 3:** Chemical composition of essential oil of *Mentha piperita*.

Compound	Area % ± SEM	Library/ID	LRI	AI	SI/MS
1	0.73 ± 0.23	α-Pinene	930	932	97
2	0.22 ± 0.012	Sabinene	965	969	91
3	0.72 ± 0.22	β-Pinene	968	974	90
4	0.40 ± 0.32	β-Myrcene	988	988	79
5	1.85 ± 0.31	Limonene	1024	1024	99
6	0.20 ± 0.021	Eucalyptol	1025	1026	98
7	0.17 ± 0.035	Linalool	1094	1095	72
8	1.35 ± 0.34	Isopulegol	1143	1145	98
9	9.48 ± 0.72	Menthone	1149	1148	95
10	8.36 ± 0.98	Isomenthone	1158	1158	97
11	67.98 ± 1.53	Menthol	1165	1167	91
12	0.46 ± 0.21	α-Terpineol	1187	1186	72
13	0.40 ± 0.17	Pulegone	1233	1233	97
14	0.49 ± 0.23	2-Hexenyl isovalerate	1243	1241	90
15	0.85 ± 0.16	Piperitone	1251	1249	96
16	2.37 ± 0.61	Menthyl acetate	1291	1294	91
17	0.23 ± 0.17	β-Bourbonene	1377	1387	93
18	0.58 ± 0.16	β-Caryophyllene	1404	1417	99
19	0.69 ± 0.15	Germacrene	1467	1484	96
20	0.11 ± 0.022	β-Germacrene	1548	1559	80
	**97.64**				

RT: Retention Time on HP-5MS column. LRI: Linear Retention Index on HP-5MS column, experimentally determined using homologous series of C7-C30 alkanes. AI: Arithmetic Index (Adams, 2007) SI/MS: Similarity Index/Mass Spectrum (NIST, 2011 Database)

Several constituents fell within the terpene fraction: isomenthone 9.48%; menthone 8.36%; limonene 1.85%. These results are in agreement with those previously reported for EOs of different *M*. *piperita* species [37]. Conceivably, the antibacterial activity, as well as the synergistic effect of EO, may be attributed to the high percentage of oxygenated monoterpenes, which represent the major components of the EO. Trombetta *et al*. [57] demonstrated that monoterpenes contained in the EOs interact with model membranes and that their antimicrobial effect may be attributed to a damage sustained by the microbial lipid membrane fraction. The Gram-negative outer membrane has a strong negative charge conferred to it by the lipopolysaccharide, which is connected to lipid composition and to the net surface charge of the microbial membrane. The lower synergistic effect observed on yeasts may be attributed to a negative interaction with the antifungal drugs.

## Conclusions

This paper describes a study regarding the association of *M*. *piperita* EO with several antimicrobial agents against a large panel of bacteria and fungi strains. Gentamicin and ampicillin were chosen as antibacterial agents, whereas amphotericin B, fluconazole and miconazole were chosen as antifungals. On the whole, a synergism between *M*. *piperita* EO and antimicrobials was found. The FIC indices for the association of gentamicin and *M*. *piperita* EO indicate, indeed, a very strong synergistic mode of action for all tested Gram-positive and Gram-negative strains. As a consequence, the combination of these two compounds allows for a significant reduction of the amount of gentamicin needed to inhibit bacteria strains. For example, a 33-fold reduction for gentamicin for *Bacillus cereus* ATCC 10876 (FICI = 0.08), a 50-fold reduction for *Bacillus subtilis* ATCC 6633 (FICI = 0.07) and 4-fold reduction for *Staphylococcus aureus* ATCC 43300 (methicillin resistant *Staphylococcus aureus*) (FICI = 0.30) were observed. Mixtures of ampicillin and *M*. *piperita* EO show marked synergistic effects against *Escherichia coli ATCC* 25922 (FICI = 0.08) and *Bacillus subtili*s ATCC 6633 (FICI = 0.13). Conversely, no evident synergistic effect was observed for ampicillin (FICI = 0.55) against Gram-negative strains as *Acinetobacter baumannii* ATCC 19606, *Klebsiella pneumoniae* ATCC 19833, and *Pseudomonas aeruginosa* ATCC 27853. The results against Gram-negative bacteria as *Klebsiella pneumoniae* and *Pseudomonas aeruginosa* are of particular interest as these bacteria are difficult to treat with commonly employed antibacterial drugs. Generally, a synergistic effect was also observed against yeast strains, although it was less evident than against bacteria. This result may conceivably depend on the poor interaction between the EO with azoles and amphotericin B. Further investigation would allow a more complete understanding of the antimicrobial potential of this association and may be useful for the preparation of new agents for the cure of infections caused by these important pathogens.
